# Epidemiology of amyotrophic lateral sclerosis: an update of recent literature

**DOI:** 10.1097/WCO.0000000000000730

**Published:** 2019-08-12

**Authors:** Elisa Longinetti, Fang Fang

**Affiliations:** Department of Medical Epidemiology and Biostatistics, Karolinska Institutet, Stockholm, Sweden

**Keywords:** amyotrophic lateral sclerosis, epidemiology, global perspective, increasing incidence

## Abstract

The cause of amyotrophic lateral sclerosis (ALS) remains unknown for most of the patients with the disease. Epidemiologic studies can help describe disease burden and examine its potential risk factors, providing thereby evidence base for future mechanistic studies. With this review, we aimed to provide a summary of epidemiologic studies published during the past 18 months, which studied the incidence and risk factors for ALS.

**Recent findings:**

An increasing incidence and prevalence of ALS continue to be reported from different parts of the world. Several previously studied risk factors are confirmed as causally related to ALS by Mendelian randomization analysis. The previously known prognostic indicators for ALS appear to be the same across populations.

**Summary:**

Provided with the increasing number of patients diagnosed with ALS and the improved societal awareness of the disease, more resources should be allocated to the research and care of ALS. Population-based studies, especially population-based disease registers, should be the priorities in ALS research, and more data from outside Europe are needed in gaining a better global perspective of the disease.

## INTRODUCTION

The cause of amyotrophic lateral sclerosis (ALS) remains today unknown for most of the patients with the disease. Epidemiologic studies can help describe disease burden and examine its potential risk factors, providing thereby evidence base for future mechanistic studies. With this review, we aimed to provide a summary of epidemiologic studies published during the past 18 months, which studied the incidence and risk factors of ALS. 

**Box 1:**
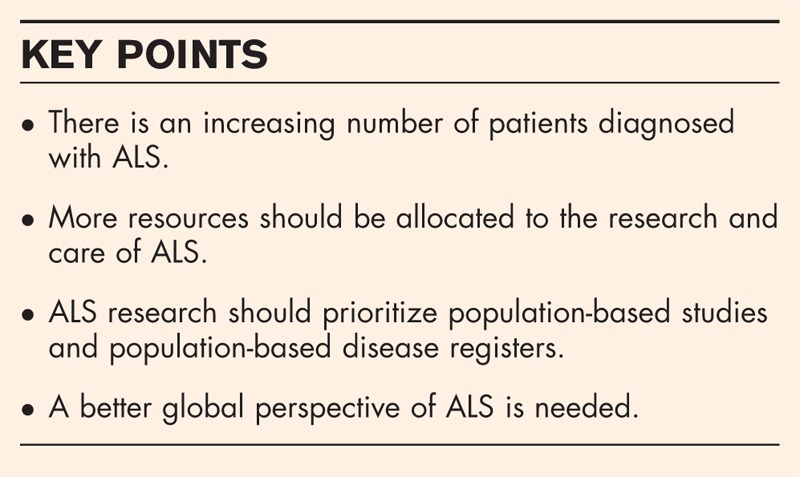
no caption available

## INCIDENCE

Incidence studies are important, not only in terms of gaining knowledge of disease burden but also to compare if and how the disease burden differs across populations of different characteristics. Recent studies reported an incidence of ALS between 0.6 and 3.8 per 100 000 person-years [1^▪^,^2^,^3^,4^▪^,^5^,^6^,^7^^▪▪^,^8^]. In Europe, the incidence of ALS is higher, ranging from 2.1 to 3.8 per 100 000 person-years [1^▪^,^2^,^3^,^7^^▪▪^]. Using information from population-based motor neuron disease registers, the incidence of ALS was 3.8 per 100 000 person-years in Stockholm (2014) and Scotland (2015–2017) [1^▪^,^7^^▪▪^]. Based on an extensive effort of information collection through different sources, a slightly lower incidence of ALS, 2.1 per 100 000 person-years during 2000–2015 in Nordland, Norway [3] and 2.8 per 100 000 person-years during 2002–2014 in Friuli-Venezia Giulia, Italy [2] was reported.

Few studies have also been published from outside Europe. One study from South Korea and one study from Beijing, China reported ALS incidence as 1.2 (2011–2015) and 0.8 (2010–2015), per 100 000 person-years, respectively [4^▪^,^6^]. The study from Korea was based on the Korean National Health Insurance Service data, whereas the study from Beijing was based on patient visit record of the Peking University Third Hospital and the census report of the Beijing Oriental Rain ALS care center. Because it is difficult to define the catchment area of the two clinical centers, it is uncertain whether the reported incidence in the latter study represents the true incidence of ALS in Beijing. Nevertheless, the relatively low incidence reported in the Korean peninsula can probably not be attributed to ascertainment bias alone and might indeed suggest a difference in ALS incidence between Asian and non-Asian populations. One speculation for a lower incidence of ALS in Asia is the lower prevalence of known ALS genes in Asian populations, as shown among patients with ALS in South Korea [9,10]. Through identifying patients with ALS from all neurologic centers and hospitals of three provinces, Turgut *et al.*[5] reported an incidence of 1.9 per 100 000 person-years during 2006–2010 in the Trace region, Turkey. Using public health insurance data, Rose *et al.*[8] reported a relatively low incidence of ALS as 0.6 per 100 000 person-years during 2003–2014. It would be interesting to understand further potential explanations for such low incidence.

In addition to the noted geographical differences, the incidence of ALS appears to be rising according to most of the publications that did test for a trend [3,5,7^▪▪^]. Leighton *et al.*[7^▪▪^] reported an increase of 36% in ALS incidence during a 25-year period in Scotland and hypothesized that the increasing incidence might be because of both improved survival rate of competitive diseases and ALS ascertainment. An increasing ALS incidence was also noted during 2006–2010 in the Thrace region, Turkey [5], but not in the Nordland county of Norway, where the incidence appeared to be stable during 2000–2015 [3]. However, because this study included only 74 patients during the entire study period, it remains a concern whether it had sufficient statistical power in detecting such a trend [3].

## PREVALENCE

Recent population-based studies reported a prevalence of ALS between 4.1 and 8.4 per 100 000 persons [1^▪^,^2^,^3^,4^▪^,^5^,^7^^▪▪^,^8^,^11^–^13^]. A slightly increasing prevalence of ALS has been suggested in most of these studies that have reported prevalence over different years [1^▪^,4^▪^,^7^^▪▪^,^8^,^11^], although the increase was not significant in the studies that tested for prevalence increase [2,13]. In the United States, for example, using three administrative healthcare data sources and capture–recapture methodology, Nelson *et al.*[11] reported an ALS prevalence of 3.7 per 100 000 in 2002, 4.4 per 100 000 in 2003, and 4.8 per 100 000 in 2004. Mehta *et al.*[12] reported a prevalence of 5.2 per 100 000 in 2015 according to the US National ALS Registry, which was similar to the prevalence of 5.0 per 100 000 as reported in 2014. In contrast to findings of population-based studies, Bhattacharya *et al.*[14] reported an ALS prevalence of 11.8 per 100 000 in 2011 through investigating a subset of the US population with a Medicare Advantage prescription drug plan. This high prevalence is likely because of the greater proportion of elderly people involved in the drug plan. Difference in ALS prevalence by ethnicity is reported in the United States, for example, in the study using the National ALS Registry the prevalence of European-American ALS patients was found to be more than double the prevalence of African-American ALS patients (5.4 versus 2.3 per 100 000) [12].

## SEX

Male sex has since long being considered a risk factor for ALS [15]. Recent studies have reported a male-to-female ratio between 1 and 2 [2,3,4^▪^,^5^,^6^,^7^^▪▪^,^11^–^14^,^16^,^17^,18^▪^,^19^–^28^], except for a report from Africa, whereas the male-to-female ratio was reported as high as 2.9 [29] and our recent study on the basis of the Swedish Motor Neuron Disease Registry, which reported a male-to-female ratio of around one in Stockholm, Sweden [1^▪^]. Overall, these data do not yet suggest a decreasing male-to-female ratio in ALS incidence, which has been proposed previously potentially as a result of the improved case ascertainment for women, especially elderly women, and the fact that women are nowadays more exposed to potential risk factors of ALS, such as smoking and occupational toxicants. However, the temporal trend of male-to-female ratio in ALS incidence might differ between countries, and needs to be examined further.

## AGE AT ONSET

Recent studies have shown that the mean or median age of ALS onset is between 51 and 66 years [1^▪^,^3^,4^▪^,^5^,^6^,^7^^▪▪^,18^▪^,^19^,^20^,^23^,^26^,^28^–^31^,^32^^▪▪^]. Patients in Europe have usually a later age at ALS onset compared with patients from China, Cuba, and Uruguay. For example, Dorst *et al.*[18^▪^] showed that Chinese ALS patients had a median age of ALS onset at 51 years, whereas the median age of ALS onset was around 10 years later among German ALS patients. Furthermore, the mean age at ALS onset was found to be 4–9 years smaller in Cuba and Uruguay compared with Ireland [26]. Population-based studies are more likely to include patients with the entire spectrum of disease characteristics, whereas clinic-based studies are more likely to include patients with specific characteristics (e.g., younger age at onset or slower disease progression) [33]. The greater age at ALS onset in Europe might, therefore, be partly attributable to the use of population-based design. Another predictor for age at ALS onset is whether the disease is familial or sporadic. Patients with familial ALS tend to have an earlier age of onset compared with patients with sporadic ALS [34]. This could partly be because of closer surveillance of ALS symptoms among relatives of ALS patients, leading to earlier clinical diagnosis of ALS [35]. However, Mehta *et al.*[36] showed that younger age of onset for patients with familial ALS can also be attributable to a Mendelian gene variant.

## DIAGNOSTIC DELAY

Initial symptoms of ALS are nonspecific and may mimic symptoms of other neuromuscular diseases. Misdiagnosis, common in the early stage, can therefore delay ALS diagnosis. In addition, given the lack of valid diagnostic biomarkers [37], the diagnosis of ALS is made clinically and requires evidence of a progressive spread of symptoms [38], which takes time to demonstrate. Recent studies have reported the mean or median diagnostic delay to range between 9 and 24 months [1^▪^,^3^,4^▪^,^5^,^6^,^7^^▪▪^,^19^,^29^,^32^^▪▪^]. In addition to familial form [36], other predictors of diagnostic delay might include site of symptoms onset and sex. For example, patients with a bulbar onset were reported to be diagnosed earlier compared with patients with a spinal onset, whereas male patients were diagnosed on average sooner compared with female patients, in Beijing [6].

## AGE AT DIAGNOSIS

The mean or median age at ALS diagnosis was reported as between 54 and 69 years in recent studies [1^▪^,^2^,^3^,4^▪^,^5^,^6^,^7^^▪▪^,^16^,^19^,^20^,^26^,^30^,^32^^▪▪^]. Predictors of higher age at diagnosis are cognitive impairment and sex. Mean age at diagnosis was reported to be eight years higher among patients with cognitive impairment compared with patients without cognitive impairment [16]. Mean age at diagnosis was slightly higher among women, compared with men, in a population-based study of North-Eastern Italy [2] and in a clinical study in Tokyo, Japan [30], but not in a clinical study from Beijing, China [6].

## SITE OF ONSET

ALS onset usually manifests as weakness in the limbs (spinal onset) or difficulty in speaking or swallowing (bulbar onset). Between 58 and 82% of ALS patients have a spinal onset [1^▪^,^2^,^5^,^6^,^7^^▪▪^,18^▪^,^19^,^26^–^31^,^32^^▪▪^]. This seems to be rather consistent across different countries. For example, Dorst *et al.*[18^▪^] compared clinical characteristics of Chinese and German patients with ALS and found that the prevalence of spinal versus bulbar onset was equally distributed between these groups. Similarly, Ryan *et al.*[26] compared clinical features of ALS patients in Cuba, Uruguay, and Ireland and found no significant differences in the prevalence of spinal onset patients. Despite the overall predominance of spinal onset ALS, bulbar onset ALS might be differentially prevalent among patients of different characteristics. For example, women [1^▪^,^2^,^6^,^27^], patients with cognitive impairment [16], and the elderly patients [6] have been reported to have a higher prevalence of bulbar onset ALS, compared with men, patients without cognitive impairment, and the relatively young patients. In contrast, Qadri *et al.*[25] found the proportion of bulbar onset ALS (27–28%) to be similar between European-Americans and African-Americans. In addition to spinal onset and bulbar onset, recent studies reported that 8–23% of ALS patients might have other forms of onset [1^▪^,^2^,^7^^▪▪^,^21^,^28^], including mixed onset (spinal and bulbar; 9.9–17.1%) [2,7^▪▪^], thoracic onset (1.5–3.5%) [28], thoracic onset or dementia symptoms (17%) [1^▪^], respiratory symptoms (1.7%) [7^▪▪^], thoracic onset or respiratory symptoms (1.9%) [32^▪▪^], or cognitive change (2.1%) [7^▪▪^]. However, these estimates are less frequently reported so far.

## NONGENETIC RISK FACTORS

An increasing number of susceptibility genes have been reported, but the overall genetic contribution to ALS still appears weak [39,40]. Until recently the only established risk factors for ALS were older age and male sex, in addition to a family history of ALS. Nevertheless, epidemiologic research in the past has focused on various suspected risk factors for ALS, including lifestyle factors, body mass index, educational attainment, exposures to toxicants, virus infections, and comorbid conditions [15,41–43]. Using summary statistics generated from various genome-wide association studies, several studies have used Mendelian randomization analysis, which has the potential to investigate causal relationship between a risk factor and a disease, avoiding common methodological problems of observational studies, such as confounding and reverse causation, to assess the role of the proposed factors for ALS. Interestingly, a causal relationship was indeed suggested between some of the studied risk factors, including blood lipid levels [44,45^▪▪^], smoking [45^▪▪^,^46^], physical activity [45^▪▪^], and educational attainment [45^▪▪^], and ALS risk. Further efforts are needed in understanding the underlying mechanisms linking together such factors and ALS.

Other recent epidemiologic studies focused on occupational exposure, dietary habits, and physical fitness. In three Danish population-based studies using complete employment history, ALS was linked to diesel exhaust [47], lead [17,48], work-related intense physical activity [17], and specific occupations including agriculture, hunting, forestry, fishing, and construction work [17]. Similarly, a multicenter population-based control study found a positive association of diesel exhaust, silica, organic dust, and extremely low-frequency magnetic fields and electric shocks with ALS risk [24,49]. Long-term exposure to air pollution has also been suggested as a risk factor for ALS [31].

Although it is important to study dietary factors, they are challenging to measure. Korner *et al.*[27] did not find evidence for an association between dietary habits and ALS. However, this study was limited by the use of spouses, hospital staff, and acquaintances as control group. Using a sample of healthy individuals as control group, and with a larger sample size, Pupillo *et al.*[32^▪▪^] found evidence that certain foods and nutrients (red and processed meat, animal protein, and sodium, zinc and glutamic acid) were associated with a higher risk of ALS, whereas others (coffee, tea, whole bread, raw vegetables, and citrus fruits) might be associated with a lower risk of ALS. No association was found between alcohol consumption and ALS [28,32^▪▪^]. Concerning physical fitness, in a large longitudinal cohort of Swedish men, low muscle strength, low BMI, and low erythrocyte volume fraction at 17–20 was related to a lower risk of ALS at early age [50].

## SURVIVAL AND PROGNOSTIC FACTORS

The survival of ALS is variable. About 10% of ALS patients have a slow form of the disease with a survival of 10 years or longer. The vast majority of ALS patients have however a much more limited survival after diagnosis. Recent studies reported a mean or median survival time from symptoms onset to death or invasive respiratory support as between 24 and 50 months [1^▪^,^3^,4^▪^,^16^,18^▪^,^20^,^23^,^25^,^26^,^29^,^30^]. Tracheostomy is commonly used as an alternative endpoint for death because the survival of ALS patients is greatly extended once the tracheostomy is in place. Benjaminsen *et al.*[3] showed in their study that the mean survival time from symptoms onset to death could be longer than five years if ALS patients were on tracheostomy. Predictors for better ALS survival include male sex, longer diagnostic delay, and attending multidisciplinary clinics. Recent studies have also reported that longer survival is associated with male sex [7^▪▪^], spinal onset [1^▪^,^3^,^5^,^7^^▪▪^,18^▪^,^19^,^27^], younger age at onset and diagnosis [18^▪^,^21^], higher baseline ALSFRS-R score [21], higher body mass index [18^▪^,^21^], and weight gain after diagnosis [30]. In contrast, respiratory or genitourinary comorbidities [14], presence of cognitive impairment and depression [16], higher concentrations of persistent organic pollutants in plasma [19], and weight loss from onset to diagnosis [23] have all been suggested to negatively affect survival of ALS patients.

The associations of different prognostic indicators with ALS survival might be universal across ethnicity groups. For example, predictors of prolonged survival appeared greatly similar when comparing Chinese ALS patients to German ALS patients [18^▪^]. The median survival time of African patients with ALS (14 months from diagnosis) were found to be in agreement with data from Western patients [29]. This is in contrast to the previous studies suggesting that African patients with ALS have a better prognosis compared with Western patients [51–53]. The representativeness of ALS patients enrolled in different studies might to a varying extent explain the observed differences of survival by ethnicity. For example, there is a clear dominance of male (male-to-female ratio 2.9) and young patients in the African study [29].

## CONCLUSION AND IMPLICATIONS

In summary, the literature during the past 18 months has contributed importantly to our understanding of the epidemiology of ALS, especially in terms of disease burden in populations outside Europe and potential risk factors that are causally related to ALS. High-quality population-based studies or population-based disease registers are clearly needed from outside Europe to gain a better estimate of the disease burden and understanding of ALS risk factors globally. Provided with the increasing number of patients diagnosed with ALS and the improved societal awareness of the disease, more resources should be allocated to ALS research. International collaborative efforts on not only research but also care of ALS are likely to maximize the efficacy of research and quality of healthcare provided for patients with ALS.

## Acknowledgements

None.

### Financial support and sponsorship

The work was funded by the Swedish Research Council (Grant No.: 2015-03170), the Ulla-Carin Lindquist Foundation, and the Karolinska Institutet (Senior Researcher Award to F.F.).

### Conflicts of interest

There are no conflicts of interest.

## REFERENCES AND RECOMMENDED READING

Papers of particular interest, published within the annual period of review, have been highlighted as:

▪ of special interest
▪▪ of outstanding interest
